# Identification of *Phakopsora pachyrhizi* Candidate Effectors with Virulence Activity in a Distantly Related Pathosystem

**DOI:** 10.3389/fpls.2016.00269

**Published:** 2016-03-08

**Authors:** Sridhara G. Kunjeti, Geeta Iyer, Ebony Johnson, Eric Li, Karen E. Broglie, Gilda Rauscher, Gregory J. Rairdan

**Affiliations:** DuPont Experimental StationWilmington, DE, USA

**Keywords:** Asian soybean rust, soybean, effectors, virulence

## Abstract

*Phakopsora pachyrhizi* is the causal agent of Asian Soybean Rust, a disease that causes enormous economic losses, most markedly in South America. *P. pachyrhizi* is a biotrophic pathogen that utilizes specialized feeding structures called haustoria to colonize its hosts. In rusts and other filamentous plant pathogens, haustoria have been shown to secrete effector proteins into their hosts to permit successful completion of their life cycle. We have constructed a cDNA library from *P. pachyrhizi* haustoria using paramagnetic bead-based methodology and have identified 35 *P. pachyrhizi* candidate effector (CE) genes from this library which are described here. In addition, we quantified the transcript expression pattern of six of these genes and show that two of these CEs are able to greatly increase the susceptibility of *Nicotiana benthamiana* to *Phytophthora infestans*. This strongly suggests that these genes play an important role in *P. pachyrhizi* virulence on its hosts.

## Introduction

Soybean rust is a devastating disease that threatens soybean crops worldwide. Its effect is most pronounced in Brazil, where crop losses and extra fungicide expenses have been calculated to be in the billions of dollars (Yorinori et al., 2005). While extensive effort has been made to discover effective genetic resistance to this disease in soybean there are currently no known resistant commercial cultivars and extensive germplasm screening has not identified soy varieties that are resistant to all known rust isolates (Walker et al., 2014). These observations, plus the fact that *Phakopsora pachyrhizi* has an unusually broad host range for an obligate biotroph (Keogh, 1976; Slaminko et al., 2008), suggest that this pathogen is very adept at evading host defenses.

Many filamentous pathogens, including rusts, exert their virulence through effector proteins that are transferred into plant cells from haustoria (Garnica et al., 2014); specialized feeding structures that become embedded in host cells without breaching the plasma membrane. Fungal effectors translocate from haustoria into plant cells through a poorly-understood mechanism and then act to modulate the physiology of their host (Petre et al., 2014). Effectors were first identified as avirulence genes that triggered strong host defense responses known as ETI (effector-triggered immunity), but many effectors have since been shown to play an important virulence function for the pathogen expressing them (Kamoun, 2007). While some effectors are thought to play a role in nutrient uptake, most characterized effectors that have a demonstrated virulence activity act by suppressing host defenses (Göhre and Robatzek, 2008). There are many examples of effectors from filamentous pathogens that have been shown to suppress plant defenses. In oomycetes, Avr3a and Avrblb2 from *Phytophthora infestans* were shown to suppress plant defense responses (Bos et al., 2010; Bozkurt et al., 2011) as were a large number of predicted effectors from *Hyaloperonospora arabidopsidis* (Fabro et al., 2011). In fungi, the rice blast (*Magnaporthe oryzae*) effector AvrPiz-t suppresses immunity in rice by targeting a RING E3 ubiquitin ligase (Park et al., 2012), while corn smut (*Ustilago maydis*) Pep1 can subdue corn defense responses via the suppression of peroxidase activity (Hemetsberger et al., 2012).

Like other filamentous pathogens, rust proteins have been identified that trigger ETI in plant cells (Dodds et al., 2004; Catanzariti et al., 2006), and a number of intriguing studies have demonstrated biochemical activities attributable to secreted rust proteins (Kemen et al., 2013; Pretsch et al., 2013; Petre et al., 2015a,b) but to date no rust effectors have been clearly shown to block plant defenses or enhance pathogen virulence. In this study, we have developed an improved method to generate a cDNA library from *P. pachyrhizi* haustoria and have bioinformatically identified 35 candidate effectors. Gene expression analysis of six of these CEs showed expression patterns consistent with these genes having a role in virulence. Additionally, we were able to show that when expressed *in planta*, two of these candidate effectors were able to dramatically enhance *P. infestans* virulence on *Nicotiana benthamiana*, suggesting that these effectors are important for *P. pachyrhizi* virulence on hosts.

## Materials and methods

### Plant growth and infection conditions

*Glycine max* was grown in a growth chamber at 22°C with a 16 h photoperiod until the primary (unifoliate) leaves had fully expanded. All experiments with *P. pachyrhizi* were performed in an USDA/APHIS-approved biocontainment facility. All inoculation experiments were conducted with a GA-05, an internal *P. pachyrhizi* field isolate collected from a soybean field in Georgia in 2005.

Before inoculation, spores were suspended in an aqueous solution of 0.01% Tween 20, heat-shocked at 40°C for 5 min and mixed thoroughly; the spore concentration was then adjusted to 1 × 10^5^ with a hemocytometer. Plants were spray-inoculated with the urediniospore suspension, incubated at 100% relative humidity in the dark for 24–36 h and then transferred to a growth chamber set at 22°C, 70% RH, 16 h photoperiod.

### *P. pachyrhizi* cDNA library construction

Fifty-four infected leaves were detached 8 days following inoculation, briefly rinsed with H_2_O and transferred to a chilled blender, where they were homogenized in 100 ml of homogenization buffer (0.3 M Sorbitol, 20 mM MOPS, 0.2% PVP, 1 mM DTT, 0.1% BSA, pH 7.2) with 0.2% RNA protect solution (Qiagen). The homogenate was filtered first through Nytex 100 mesh and then through Nytex 25. The filtrate was then concentrated by centrifugation and resuspended in suspension buffer: 0.3 M Sorbitol, 10 mM MOPS, 0.2% BSA, 1 mM CaCl_2_, 1 mM MnCl_2_, and kept on ice. The resuspension was divided into six aliquots of 1 ml each and mixed with Con-A-biotin paramagnetic beads which were prepared by mixing 150 μl of 1 mg/ml Con-A-biothin, 150 μl of streptavidin paramagnetic beads and suspension buffer in a total volume of 900 μl. Con-A-streptavidin bead complex (200 μl) was added to each 1 ml aliquot of the resuspended homogenate and mixed at 4°C for 30 min. The mixture was then washed by placing tubes in magnetic stands and exchanging suspension buffer three times after beads have aggregated proximal to the stand. After the last wash was removed, the beads were suspended in 250 μl of Trizol and transferred to a glass dounce, where the collected tissue was homogenized. Trizol solution was transferred to a microfuge tube with 200 μl of chloroform, mixed and centrifuged. The aqueous phase was then transferred to a new tube and the RNA was precipitated with NaCl and isopropanol. Precipitated RNA was pelleted and resuspended in 20 μl H_2_O. This total RNA was used to make a cDNA library using the Clontech SMART directional cDNA kit according to the manufacturer's recommendations.

### Gene expression profiling

Soybean (*Glycine max*, var. Jack) plants were grown to the VC stage and then spray-inoculated with a suspension of *P. pachyrhizi* spores (GA05-1; 100 k spores/ml 0.01% Tween 20). Unifoliate leaves were collected from three replicate plants and flash frozen in liquid nitrogen at 0, 12, 24, 36, 48, 72, 96, and 168 h post-infection (hpi). Total RNA was prepared from either uninfected or infected leaf tissue using Trizol Reagent (Life Technologies #15596-026). Isolated total RNA was DNAse-treated and cDNA synthesized using the QuantiTect Reverse Transcription Kit (Qiagen #205311). Absolute quantification of transcript levels was determined by TaqMan qPCR (TaqMan Gene Expression Master Mix; Applied Biosystems #4309849). Each sample was run in triplicate on a QuantStudio™ 6 Flex Real-Time PCR System (Applied Biosystems) using cDNA generated from 200 ng of total RNA and the primers (200 nM each) and probes (100 nM) indicated in Supplementary Table 1. QuantStudio 6 and 7 Flex Software was employed for analysis. Effector transcript levels in infected plants are expressed relative to those of a *P. pachyrhizi* α-tubulin gene DN739993.1, (van de Mortel et al., 2007) that was used as an internal control for expression studies.

### Effector virulence assays

*N. benthamiana* plants were grown in a controlled environment green house at 22°C with 55% humidity at 16 h light. Agrobacteria were incubated in induction buffer (1 l MMA: 5 g MS salts, 1.95 g MES, 20 g sucrose, 200 μM acetosyringone, pH 5.6) for at least 1 h prior to infiltration into leaves as described (Bos et al., 2006).

*P. infestans* (wild-type isolated from an infested tomato field in New Castle County, Delaware) was grown on pea agar at 18°C in the dark. Two leaves of 4- to 5-week old *N. benthamiana* plants were agro-infiltrated at an OD_600_ of 0.3 with the binary vector pMAXY226 (tagged with 3x FLAG toward the C-terminus) on one half of the mid-vein and an effector cloned into pMAXY226 (tagged with 3x FLAG toward the C-terminus) into the other half of the same leaf. *P. infestans* sporangia were harvested and diluted to ~100,000 spores/ml (Kamoun et al., 1998; Schornack et al., 2010). Droplets (10 μl) of zoospores were applied onto the abaxial side of detached leaves 24 h post agro-infiltration and incubated for several days on wet paper towels in 100% relative humidity (King et al., 2014). To determine lesion size, leaves were placed on a light box and photographed. On the resulting image, the border of the lesion that was visible (dark brown region) was marked manually on digital images of standardized size. An example of a marked image is shown in Supplementary Figure 1. Lesion area within this border was then calculated with Image-Pro Analyzer (v7.0) software from these images.

Effectors CSEP-07, CSEP-08, CSEP-09, and CSEP-35 were synthesized (Genscript, Piscataway, NJ) without signal peptide and cloned into an expression vector fused with 3x FLAG tag at the 3′ end of the gene. Mature effector PexRD2 was amplified from the genomic DNA of *P. infestans* using KOD DNA high fidelity polymerase (Novagen) and was also sub-cloned into the expression vector with a 3x FLAG tag. The sequence of PexRD2 was confirmed by sequencing. All three cloned vectors were sequence-verified and transformed into *Agrobacterium tumefaciens* (AGL1 competent cells).

Agro-infiltrated *N. benthamiana* leaves were harvested at 5 days post-infiltration (dpi). Total protein extracts were prepared by grinding five leaf discs (6.0 mm each) in 1 ml radioimmunoprecipitation assay (RIPA) lysis and extraction buffer (Pierce® RIPA buffer product no. 89900; Thermo Scientific, Rockford, IL, USA) in the presence of 0.1 mM protease inhibitor HALT Protease and Phosphatase inhibitor cocktail (Thermo Scientific, no. 78442).

### Accession numbers

Sequence data from this article can be found in the GenBank data library under accession numbers KU695151–KU695185.

## Results

In order to understand *P. pachyrhizi* virulence, we sought to identify genes that encode effector proteins. Rust effectors are synthesized in and translocated into host cells from specialized structures called haustoria, so we generated and screened a cDNA library from isolated haustoria, an approach that has been successful in identifying effectors from other rust fungi (Hahn and Mendgen, 1992; Catanzariti et al., 2006).

**Figure 1:**
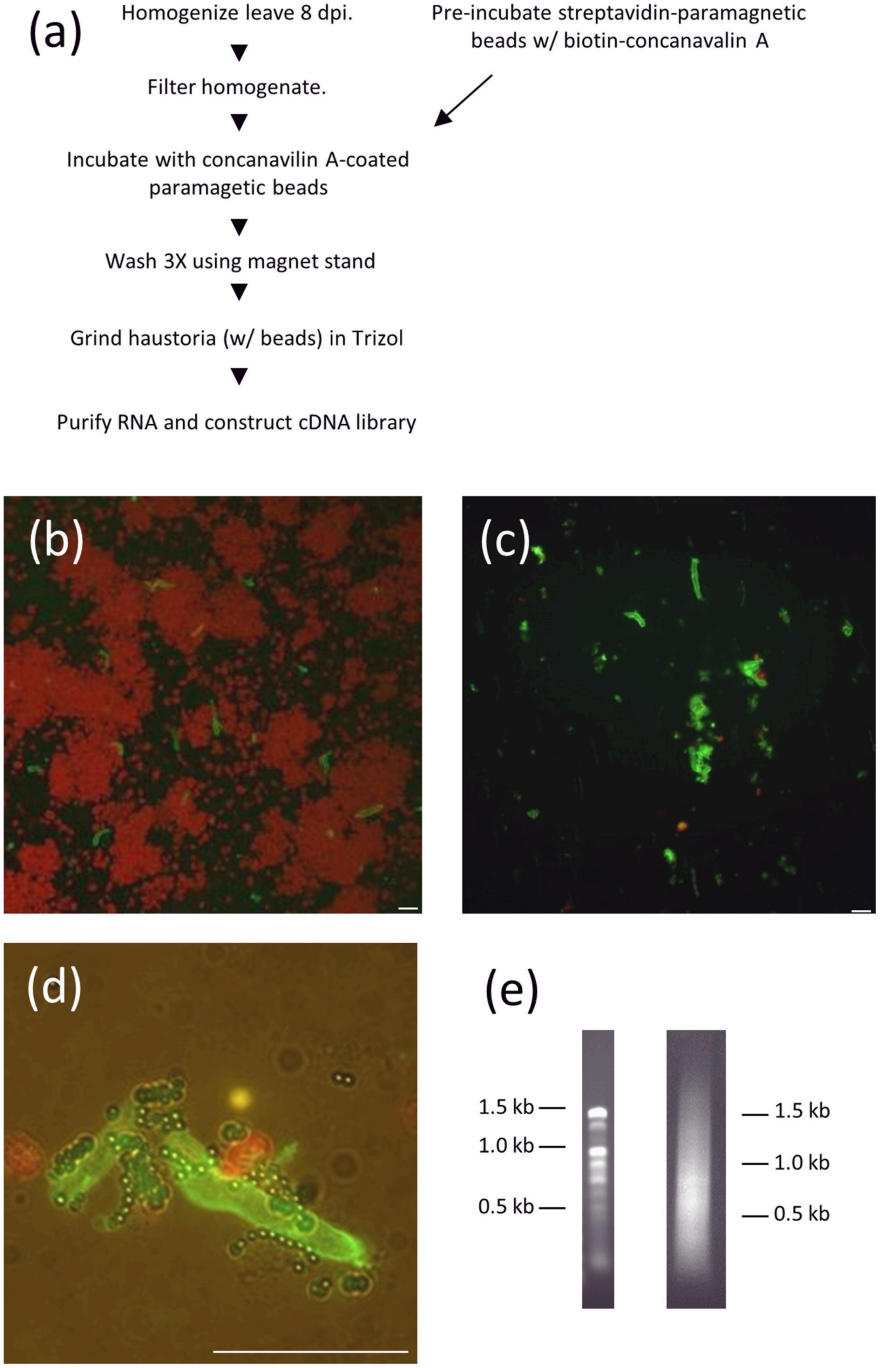
**RNA purification from isolated ***P. pachyrhizi*** haustoria. (A)** Schematic of haustorial RNA purification. **(B)** Micrograph of ConA-FITC-stained haustoria after homogenization and filtration through Nytex membrane. Red objects are chloroplasts. Bar = 10 μm **(C)** ConA-FITC stained haustoria after paramagnetic bead purification. Bar = 10 μm **(D)** Close up of ConA-FITC stained haustoria after purification showing association with paramagnetic beads. Bar = 10 μm **(E)** 1% Agarose gel separation of 1 μg haustorial RNA (left) and 0.5 μg cDNA prep (right).

We developed a new method for isolating haustorial RNA as we were initially unsuccessful in isolating RNA from haustoria using concanavalin A-conjugated beads, the method that has been successfully used to generate cDNA libraries from other rust species (Hahn and Mendgen, 1997; Catanzariti et al., 2006). Link et al. (2014) also noted difficulty in identifying high-quality RNA from *P. pachyrhizi* haustoria using Con-A sepharose beads, but were ultimately successful in generating a haustorial transcriptome using next-generation sequencing. We homogenized soybean leaves infected with *P. pachyrhizi* field isolate GA-05 8 days prior and filtered the extract through Nytex membranes. Instead of a Sepharose A column, we used streptavidin-conjugated paramagnetic beads that were then coated with biotin-concanavalin A (Figure 1A). These concanavalin A beads were then added to filtered extract of leaves that had been inoculated with *P. pachyrhizi* 8 days prior. The bound fraction of the extract was washed twice, greatly reducing chloroplast abundance (Figures 1B–D), and then RNA was extracted. We extracted 2.7 μg of high quality RNA from 54 infected leaves, and this RNA was used to construct a directional cDNA library (Figure 1E). We obtained quality sequence from 6481 clones using Sanger sequencing, which provided us with 500–900 bp of 5′ sequence for each clone. This collection of sequences was then assembled into 1944 contigs, 1633 of which were singletons. We identified 995 ORFs predicted to encode proteins of fifty amino acids or more in these 1944 contigs and these ORFs. A BLAST search identified 187 of these sequences that had a closest homolog from the plant kingdom and these were disregarded. The remaining 808 sequences were then analyzed with the SignalP algorithm to identify sequences with predicted secretory signal peptides (Emanuelsson et al., 2007). If the sequence did not encode any signal peptides, or if an ORF encoding a signal peptide was not the longest ORF for that sequence, the clone was disregarded. In addition the sequence was discarded if there was not a stop codon seen 5′ to the start codon of the largest ORF, which eliminates partial transcripts with incomplete ORFs. This left us with a collection of clones with sequences of predicted haustorially-expressed secreted proteins (HESPs). Within this collection of HESPs we found homologs of a number of previously characterized HESPs, such as hexose transporter 1, AAT1, and RTP1 (Hahn and Mendgen, 1997; Mendgen et al., 2000; Voegele et al., 2001).

While only six rust effectors have been validated to date (Petre et al., 2014), the most striking similarity of these effectors is that they do not have homology to any proteins from species outside of Pucciniales (Saunders et al., 2012; Petre et al., 2014). We thus used this as the primary criterion to identify the strongest effector candidates from *P. pachyrhizi*. We searched the HESP collection against Genbank and fully sequenced a representative clone from each contig that did not have clear homologs from any non-Pucciniales species. The predicted proteins from fully-sequenced clones were again searched against the Genbank protein database to rule out non-rust orthologs and the remaining sequences comprise our collection of *P. pachyrhizi* candidate effectors. These coding sequences are predicted to encode *P. pachyrhizi* candidate secreted effector proteins and they were thus named Pp-CSEP-01 to Pp-CSEP-35 (Supplementary Files 1, 2).

Within our collection of CEs, twenty-four are unique to *P. pachyrhizi*, while twelve have orthologs in at least one other rust species (Table 1). We also found that 31/35 of our CEs were identified in the *P. pachyrhizi* Thai1 transcriptome described by Link et al. (2014), although only fifteen of these were identified as secreted proteins in that study (Table 1). Nine of the CEs identified here were not annotated as secreted proteins in Link et al. (2014) because the assembled transcript was not full length and presumably would not have been identified as an ORF. While effectors from filamentous pathogens have been shown to evolve rapidly (Allen et al., 2004; Dodds et al., 2006; Win et al., 2007), we found that most of the *P. pachyrhizi* Thai1 homologs were highly conserved with the CEs we identified from GA-05. Only CSEP-01, CSEP-14, and CSEP-22 have clear Thai1 homologs with <90% amino acid identity. Interestingly, CEs that have homologs in other rust species are only relatively distantly related to these proteins, with no orthologs having more than 50% amino acid identity. A public EST collection generated from germinating urediniospores (Posada-Buitrago and Frederick, 2005) was searched for these sequences and only six CEs were found (Table 1). This EST collection contains over 34,000 sequences while our haustorial collection consists of <6500 clones, suggesting that the transcripts of 29/35 of these CEs are more abundant in haustoria than urediniospores. While true effectors are often strongly induced while the pathogen is in planta, it is not always the case (Dodds et al., 2004; Catanzariti et al., 2006), so we cannot exclude the transcripts that are expressed in urediniospores from our CE collection.

**Table 1:** **Candidate ***Phakopsora pachyrhizi*** effectors**.

**Pp CSEP**	**Public EST^a^**	**Orf size (AA)**	**Cysteines**	**Unique to *Pp*^b^**	**Ortholog accession number (Organism)^c^**	**Link et al. contig^d^**
Pp-CSEP-01	N	76	2	x		**5608**
Pp-CSEP-02	N	55	5	x		4757
Pp-CSEP-03	N	208	8	49	XP_003322944 (*Pgt*)	
Pp-CSEP-04	N	204	2	x		**5072**
Pp-CSEP-05	N	291	1	x		**3430**
Pp-CSEP-06	N	218	3	35	XP_003324954 (*Pgt*)	**4760**
Pp-CSEP-07	N	105	5	x		6714
Pp-CSEP-08	N	198	12	34	XP_007408015 (*Mlp*)	**3139**
Pp-CSEP-09	N	182	12	37	XP_007408015 (*Mlp*)	**8880**
Pp-CSEP-10	N	103	3	x		**5608**
Pp-CSEP-11	N	147	1	x		**7075**
Pp-CSEP-12	N	192	3	28	XP_007404648 (*Mlp*)	**3185**
Pp-CSEP-13	N	142	4	x		**1885**
Pp-CSEP-14	N	126	2	x		7139
Pp-CSEP-15	N	311	4	x		1327^†^
Pp-CSEP-16	N	270	2	46	KNF03382 (*Pst*)	**3900**
Pp-CSEP-17	N	240	4	x		2907^†^
Pp-CSEP-18	N	195	6	x		864^†^
Pp-CSEP-19	N	183	2	x		324
Pp-CSEP-20	N	327	5	x		3176^†^
Pp-CSEP-21	N	131	0	x		3969
Pp-CSEP-22	N	294	1	x		1454^†^
Pp-CSEP-23	N	134	0	x		**6204**
Pp-CSEP-24	N	312	2	x		2715^†^
Pp-CSEP-25	N	357	4	x		1326^†^
Pp-CSEP-26	N	196	7	32	XP_003332659 (*Pgt*)	
Pp-CSEP-27	Y	348	11	30	XP_003328542 (*Pgt*)	8815^†^
Pp-CSEP-28	N	321	3	x		7972^†^
Pp-CSEP-29	N	301	4	30	KNF03382 (Pst)	1256
Pp-CSEP-30	N	119	0	x		
Pp-CSEP-31	Y	213	9	x		**1525**
Pp-CSEP-32	Y	142	0	42	XP_003326807 (*Pgt*)	**3471**
Pp-CSEP-33	Y	161	8	34	XP_007403891 (*Mlp*)	
Pp-CSEP-34	Y	200	1	x		1607
Pp-CSEP-35	Y	291	20	44	KNZ58433 (*Ps*)	**4224**

a *presence of CSEP in public urediospore EST collection*.

b *x represents a CE for which orthologs could not be identified. Numbers are the percent amino acid identity of closest ortholog; numbers in bold are annotated as secreted proteins in Link et al. (2014)*.

c *accession number of closest identified ortholog, source organism in parenthesis. Pgt, Puccinia graminis f. sp tritici, Mlp, Melampsora larici-populina; Pst, Puccinia striiformis f. sp. Tritici; Ps, Puccinia sorghi*.

d *contigs in bold are annotated as secreted proteins in Link et al. (2014)*.

† *assembled transcript in Link et al. (2014) is not full-length*.

**Figure 2:**
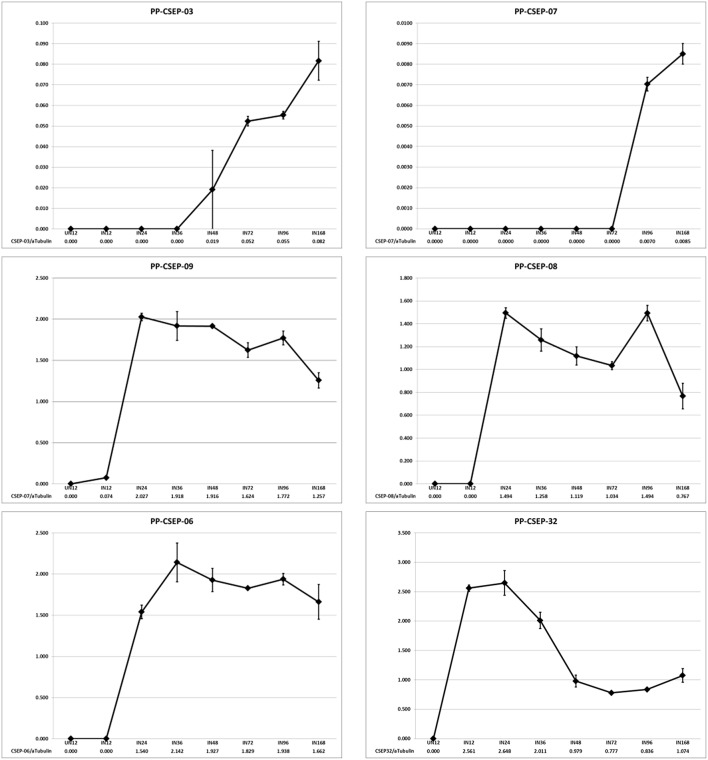
**Expression profiles of selected ***P. pachyrhizi*** candidate effectors**. RNA was harvested from soybean leaves at 12, 24, 36, 48, 72, 96, and 168 h after spray inoculation and transcript levels were quantified using qRT-PCR. RNA was also harvested from uninoculated tissue as a negative control (UN12). Error bars are standard error of the mean of three biological repeats.

We wished to determine if the expression of these CEs were induced in planta. To determine the expression patterns of these CEs we analyzed the transcript levels of a random subset of six effectors during an infection time course using qRT-PCR (Figure 2). We measured CE transcript abundance as a fraction of the abundance of *P. pachyrhizi* α-tubulin, a gene shown to be constitutively expressed in rust species (Hacquard et al., 2011). We found that in 5/6 cases the CEs were strongly induced in planta, with two primary expression patterns observed: CSEP-03 and CSEP-07 transcript levels were undetectable in early infection and induced after day 3; in contrast, CSEP-06, CSEP-08, and CSEP-09 are maximally induced by 24 h and then continue to express throughout infection. CSEP-32 is the only CE strongly expressed at 12 h post-inoculation and is maximally induced at 24 h after which expression decreases. This also is consistent with the library data, as CSEP-32 is the only of these six CEs that could be found in the Posada-Buitrago EST collection (Posada-Buitrago and Frederick, 2005).

True effectors facilitate pathogen infection and we used a *N. benthamiana/P. infestans* pathosystem to detect virulence phenotypes of our CEs. This pathosystem was previously used to demonstrate the increased pathogen growth in *N. benthamia* transiently expressing *P. infestans* effector PexRD2 (King et al., 2014). We chose this system to assay *P. pachyrhizi* effectors because, like *P. pachyrhizi, P. infestans* is a hyphal pathogen and may be susceptible to the same plant defense responses. In addition, *N. benthamiana* is well-known for its high level of foreign protein expression (Goodin et al., 2008) and *P. infestans* only grows modestly on this host which allows for increased virulence to be more readily measured than a better-adapted plant-pathogen interaction.

**Figure 3:**
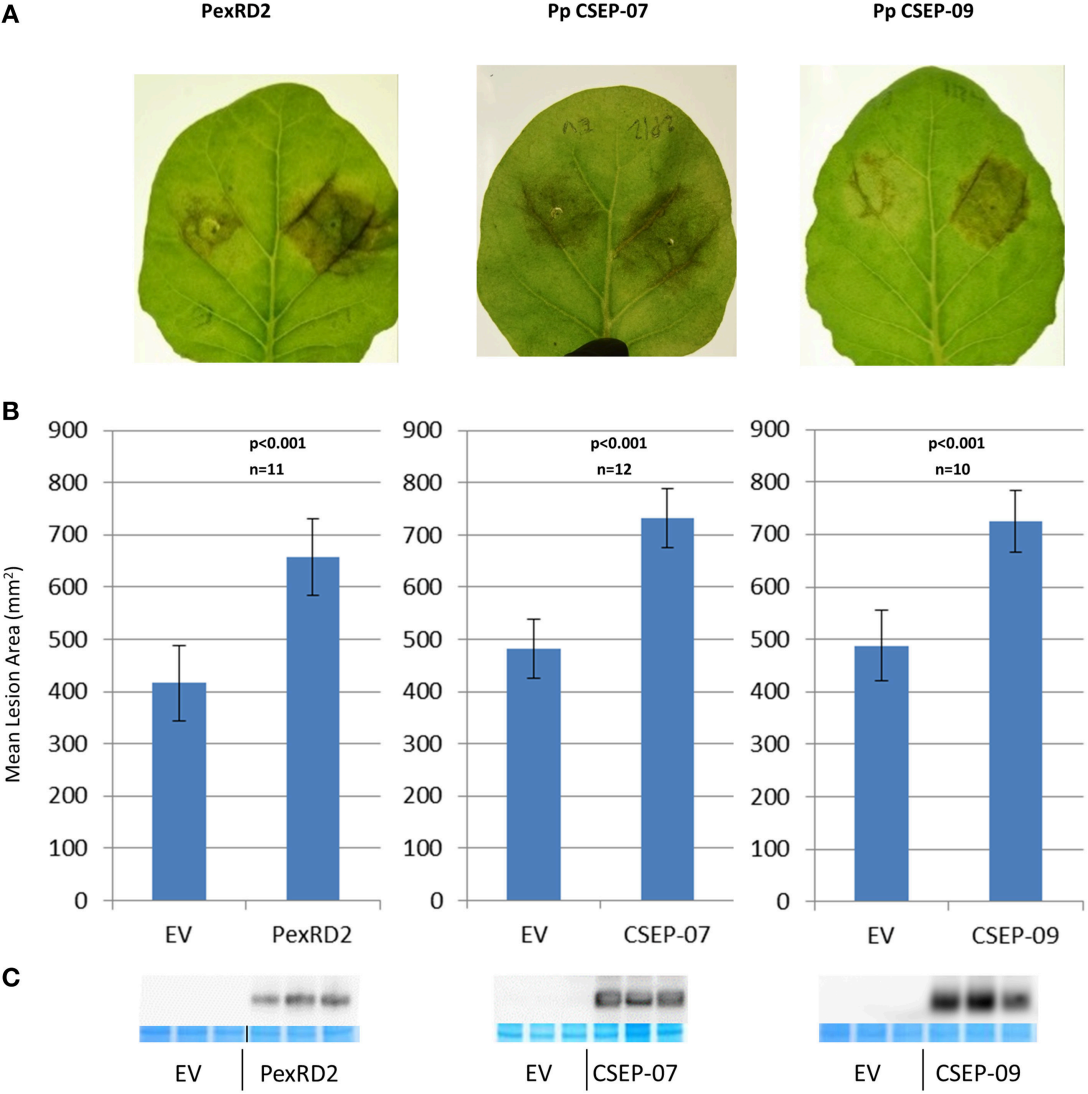
**Virulence phenotypes of ***P. pachyrhizi*** candidate effectors CSEP-07 and CSEP-09 (A) Representative leaves infiltrated agro-infiltrated with empty vector (left half of leaf) or indicated gene (right half of leaf), followed by inoculation with ***P. infestans*** zoospores**. Images were taken at 7 days after zoospore inoculation. **(B)** Quantification of *P. infestans* lesion area 7 days after inoculation. *P*-value is from a two-tailed paired Student's *t*-test. Results are an average of at least 10 inoculated leaves. **(C)** Immunoblot showing expression of indicated proteins from three plants that were agro-infiltrated but not inoculated with *P. infestans*. For each CE, protein was extracted from three independently infiltrated plants and immunoblotted with α-FLAG antibody. Directly below, protein from the same extraction was also loaded on a second gel and stained to demonstrate equal loading. This experiment was repeated twice with similar results.

We expressed *P. pachyrhizi* CEs via agrobacterium-mediated transient expression. CEs were cloned into a T-DNA vector that expresses the mature (signal peptide-truncated), epitope-tagged effector under the regulation of the strong double-Mirabilis Mosaic Virus promoter (Dey and Maiti, 1999). Each leaf was infiltrated with an effector and empty-vector control side-by-side. Twenty-four hours after infiltration *N. benthamiana* leaves were detached and both halves of the leaf were drop-inoculated with *P. infestans* zoospores and then incubated at room temperature as described by King et al. (2014). Four CEs, CSEP-07, CSEP-08, CSEP-09, and CSEP-35, were chosen to test. Expression of two of these CEs, CSEP-07, and CSEP-09 showed a dramatic increase of *P. infestans* growth at s7 days post-inoculation, and these two effectors were chosen to study in greater detail. In further experiments, *P. infestans* growth was quantified 7 days after inoculation by photographing leaves and analyzing them with ImagePro Analyzer v7.0. Figure 3A shows representative infected leaves expressing CSEP-07, CSEP-09, and *P. infestans* PexRD2 as a positive control. The area infected in each lesion was calculated and plotted in Figure 3B. A clearly statistically significant increase in *P. infestans* growth was seen in tissue expressing these three effectors compared to controls; a paired student's *t*-test calculated the *p*-value to be <0.001 in each case. To confirm that the effectors being tested were expressed in the plant tissue, protein was extracted from plants infiltrated at the same time and immunoblotted using the α-FLAG antibody (Figure 3C). No macroscopic cell death phenotype was seen at the time of protein harvest (5-dpi).

## Discussion

A biotrophic pathogen's ability to infect and colonize a host is at least partially a function of the collection of the effectors that it expresses during its life cycle. Identifying and characterizing the effectors of *P. pachyrhizi* is an important step toward understanding how this pathogen infects important crops like soybean and causes devastating economic consequences. We identified 35 *P. pachyrhizi* coding sequences that meet our strict criteria for effector candidates; a secretory signal peptide and the absence of any clear homologs outside of Pucciniales. Over half of predicted effectors are smaller than 200 amino acids, and over 80% are <300. In addition, most transcripts encoding these CEs were not found in a germinating urediniospore EST collection, suggesting haustorial enrichment.

Three rust genomes have been sequenced to date, *Puccinia graminis* f. sp. *tritici, Puccinia stiiformis* f. sp. *tritici, Melampspora larici-populina* (Duplessis et al., 2011; Cantu et al., 2013), and each has been shown to have a large number of families of short, secreted proteins that are presumed to be effectors important for pathogenesis. In addition, there have been thorough transcriptomic analyses of coffee rust, bean rust, and soybean rust haustoria (Fernandez et al., 2012; Link et al., 2014). Each of these studies has identified many more candidate effectors than we present here. This is partly because our criteria for what qualifies as a candidate effector (CE) is stricter than in these reports, and partly because the next-generation sequencing methods used in these studies provides a much deeper sampling of transcript diversity.

When we initially tried to purify haustoria from infected leaves using a column of concanavalin A-conjugated sepharose B we found that the resulting RNA was too degraded to make cDNA libraries of the desired quality. We consequently modified our haustorial purification methodology to utilize streptavidin-conjugated paramagnetic beads that were pre-bound to biotin-concanavalin A and then used to bind *P. pachyrhizi* haustoria. Paramagnetic bead capture resulted in a greatly shortened window of time between homogenization of the infected leaves and extraction of RNA because of very short washing steps and because it was not required to liberate the haustoria from the beads before they were used for RNA extraction.

Two of our effector candidates, Pp CSEP-07 and Pp CSEP-09, are able to increase the virulence of *P. infestans* on *N. benthamiana* expressing them. Since these two effectors have such dramatic phenotypes, counteracting their biochemical activity may significantly impair the virulence of *P. pachyrhizi* and doing so may provide a method for controlling the disease. The observation that a rust effector can enhance the virulence of *P. infestans* also suggests that at least some plant defenses to rust and *P. infestans* are shared, and it is likely that they are counteracted by both fungal and oomycete pathogens despite these pathogens being very distantly related to each other.

## Author contributions

SK, GI, EJ, and EL performed the experiments KB, GR, and GJR conceived and supervised the experiments and analyzed results. SK and GJR wrote the manuscript.

### Conflict of interest statement

The authors declare that the research was conducted in the absence of any commercial or financial relationships that could be construed as a potential conflict of interest. This research is protected by US patent US20140283207 A1.

## Supplementary material

Supplementary Figure 1 **Examples of the region identified to mark lesion area**. The border of the lesion was manually marked on a digital image of an infected leaf on a lightbox. The border is defined as the junction between the dark brown region of the leaf and the greener, unaffected region.

Supplementary Table 1 **Primers for qRT-PCR experiments**.

Supplementary File 1 **CSEP nucleotide sequences**.

Supplementary File 2 **CSEP amino acid sequences**.
