# Supplementary figures and images for: Identification of Phakopsora pachyrhizi Candidate Effectors with Virulence Activity in a Distantly Related Pathosystem
Source: Front Plant Sci. 2016 Mar 8;7:269. doi: 10.3389/fpls.2016.00269 (PMC4781881; doi:10.3389/fpls.2016.00269)

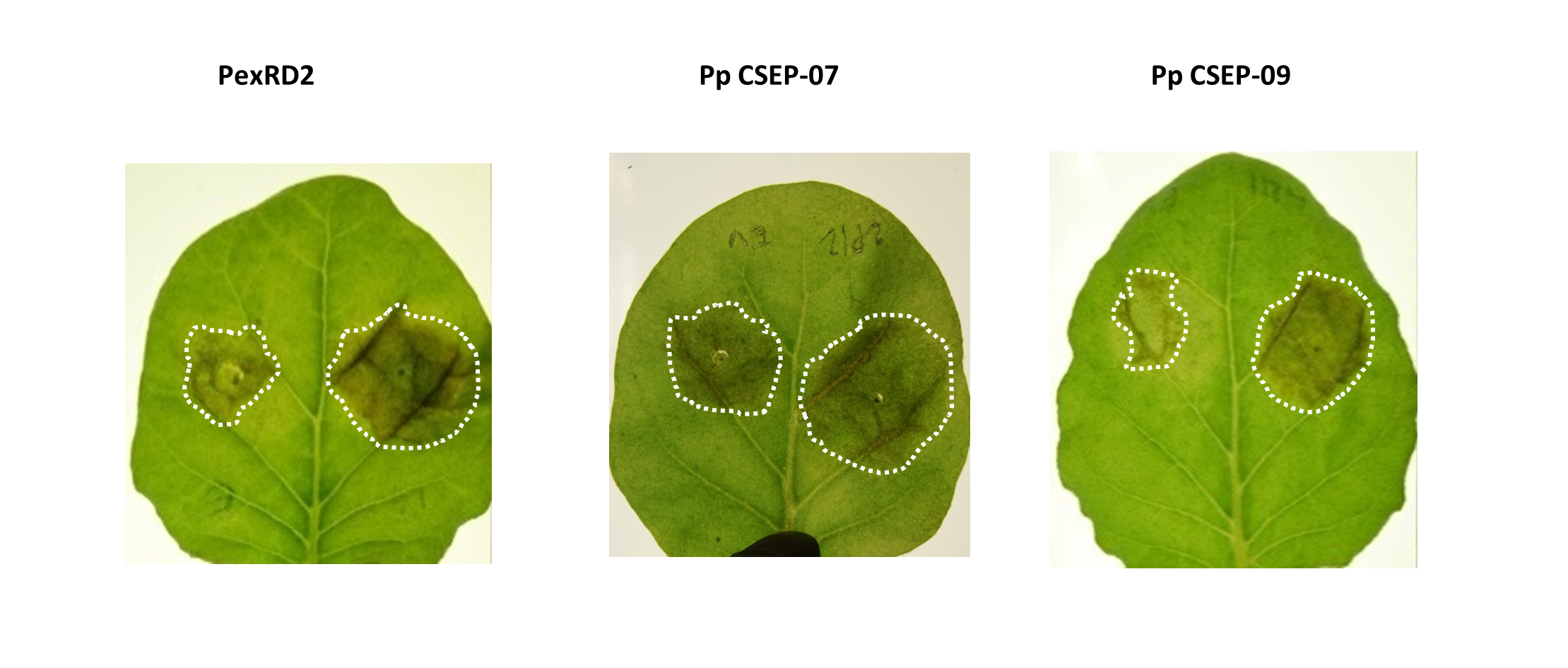

Supplement: Supplementary Figure 1 — Examples of the region identified to mark lesion area. The border of the lesion was manually marked on a digital image of an infected leaf on a lightbox. The border is defined as the junction between the dark brown region of the leaf and the greener, unaffected region. [file Image1.TIF]
