# Supplementary material for: Identification of Phakopsora pachyrhizi Candidate Effectors with Virulence Activity in a Distantly Related Pathosystem
Source: Front Plant Sci. 2016 Mar 8;7:269. doi: 10.3389/fpls.2016.00269 (PMC4781881; doi:10.3389/fpls.2016.00269)
Supplement: Supplementary Table 1 — Primers for qRT-PCR experiments. [file Table1.DOCX]

Supplementary Table 1.

| Primer/probe | Sequence 5' - 3' | Description |
| --- | --- | --- |
| CSEP-03Forw1 | AAACCCTCGCACAGGAAAAA | Forward primer for CSEP-03 amplification |
| CSEP-03Rev1 | TGAAAATCGCGGCCATCTA | Reverse primer for CSEP-03 amplification |
| CSEP-03Probe1 | 6FAM-CGTCTACGTAAAGGTC-MGBNFQ | Probe for detection of CSEP-03 amplification product of CSEP-03Forw1 and CSEP-03Rev1  (Fragment size= 57 bp) |
| CSEP-06Forw1 | CGAGTGGCTTCCCTGACATC | Forward primer for CSEP-06 amplification |
| CSEP-06Rev1 | CATGTTTCCCCGGTTGGA | Reverse primer for CSEP-06 amplification |
| CSEP-06Probe1 | 6FAM-AAATTCGACATGAAGCTT-MGBNFQ | Probe for detection of CSEP-06 amplification product of CSEP-06Forw1 and CSEP-06Rev1  (Fragment size= 61 bp) |
| CSEP-07Forw1 | GATAGGCGTGATGTCCTCGAA | Forward primer for CSEP-07 amplification |
| CSEP-07Rev1 | CGATGCCGTGGATTGGAT | Reverse primer for CSEP-07 amplification |
| CSEP-07Probe1 | 6FAM-CTGTTAGACCAAAGCC-MGBNFQ | Probe for detection of CSEP-07 amplification product of CSEP-07Forw1 and CSEP-07Rev1  (Fragment size= 58 bp) |
| CSEP-08Forw1 | GGGCCCATGCACAAGTTTTA | Forward primer for CSEP-08 amplification |
| CSEP-08Rev1 | CAATCGCTGGATCGTTTATATTGA | Reverse primer for CSEP-08 amplification |
| CSEP-08Probe1 | 6FAM-AGCGTGTGGAGATTG-MGBNFQ | Probe for detection of CSEP-08 amplification product of CSEP-08 Forw1 and CSEP-08 Rev1  (Fragment size= 62 bp) |
| CSEP-09Forw1 | GGAAACACCCCCACCAAAGT | Forward primer for CSEP-09 amplification |
| CSEP-09Rev1 | CCGGAGCAGACGAGTTGTTTA | Reverse primer for CSEP-09 amplification |
| CSEP-09Probe1 | 6FAM-ACTGCTCAGCCTCAG-MGBNFQ | Probe for detection of CSEP-09 amplification product of CSEP-09Forw1 and CSEP-09Rev1  (Fragment size= 58 bp) |
| CSEP-33Forw1 | GGCGGAACCCAGTTTTTGA | Forward primer for CSEP-33 amplification |
| CSEP-33Rev1 | TTGGGCACATTAATTTGAAAGGT | Reverse primer for CSEP-33 amplification |
| CSEP-33Probe1 | 6FAM-TGCCAGAGGAAACAT-MGBNFQ | Probe for detection of CSEP-33 amplification product of CSEP-33Forw1 and CSEP-33Rev1  (Fragment size= 59 bp) |
| αTubulinForw1 | CCAAGGCTTCTTCGTGTTTCAT | Forward primer for amplification of *P.pachyrhizi* internal control α-tubulin gene |
| αTubulinRev1 | AGAGCGCCAAACCCTGAAC | Reverse primer for amplification of *P.pachyrhizi* internal control α-tubulin gene |
| αTubulinProbe2 | VIC-CGTTTGGAGGCGGAC-MGBNFQ | Probe for detection of *P. pachyrhizi* α-tubulin amplification product of α-tubulinForw1 and α-tubulinRev1 (Fragment size= 59 bp) |
